# A Case of Popliteal Venous Aneurysm Diagnosed after Sudden Cardiac Arrest

**DOI:** 10.3400/avd.cr.23-00097

**Published:** 2024-03-13

**Authors:** Daigo Shinoda, Koichi Yuri, Atsushi Miyagawa, Nobu Yokoyama

**Affiliations:** 1 Department of Cardiovascular Surgery, Tokyo Metropolitan Bokutoh Hospital, Tokyo, Japan

**Keywords:** popliteal venous aneurysm, sudden cardiac arrest, pulmonary embolism

## Abstract

A popliteal venous aneurysm (PVA) is a rare vascular disorder. We report a case of PVA discovered through further evaluation of sudden cardiac arrest (CA) caused by a pulmonary embolism (PE). It is well-known that PVA causes PE; however, there are few reports of PVA causing CA. A tangential aneurysmectomy and lateral venorrhaphy were performed. The patient’s postoperative course was uneventful. When contrast-enhanced computed tomography is performed to search for the cause of CA, PVA should be considered and thus, screening below the knee is recommended. At 1-year follow-up, there were no complications.

## Introduction

Pulmonary embolism (PE) is a critical cause of cardiac arrest (CA), accounting for at least 4%–10% of CA cases, and is associated with an unfavorable prognosis.^1)^ PE and deep venous thrombosis (DVT) are considered a series of clinical conditions; DVT may be due to a venous aneurysm. In contrast to varicose veins caused by valvular dysfunction, venous aneurysms are uncommon. A popliteal venous aneurysm (PVA) can lead to PE; however, there are few reports of PVA causing CA. The reported risk of recurrent PE is high in patients with PVA, even in those receiving anticoagulation treatment; therefore, surgical intervention is recommended.^2)^ Here, we report the successful treatment of PVA discovered by further evaluation of sudden CA caused by PE.

## Case Report

**Figure 1:**
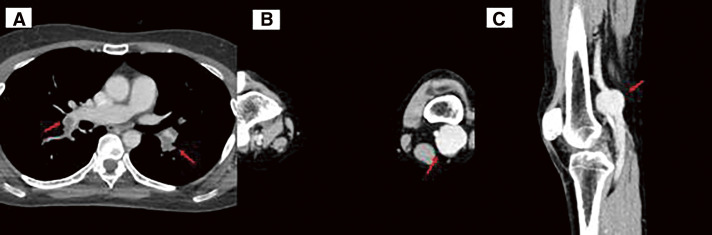
Fig. 1 Preoperative contrast-enhanced CT. (**A**) CT reveals a submassive PE. The right side is occluded from the superior trunk pulmonary artery bifurcation, and the left side is occluded from the lingular artery bifurcation. (**B**) CT reveals a saccular PVA. (**C**) The PVA has a maximum diameter of 32 mm and a length of 30 mm. CT: computed tomography; PE: pulmonary embolism; PVA: popliteal venous aneurysm

**Figure 2:**
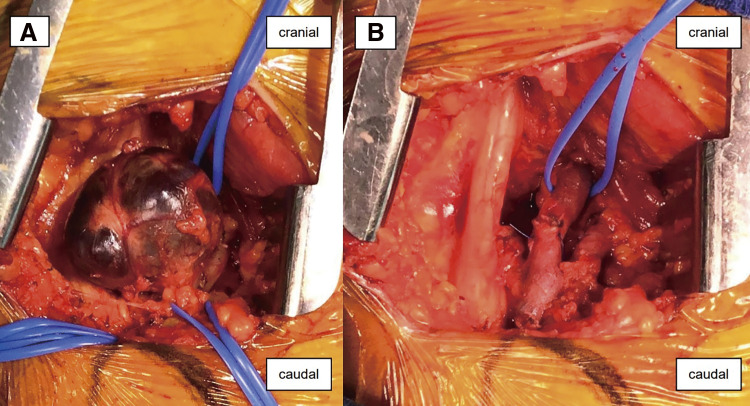
Fig. 2 Surgical findings. (**A**) Intraoperative view of the PVA shows a sharp demarcation from the normal vein segment. (**B**) Tangential aneurysmectomy and lateral venorrhaphy were performed. PVA: popliteal venous aneurysm

**Figure 3:**
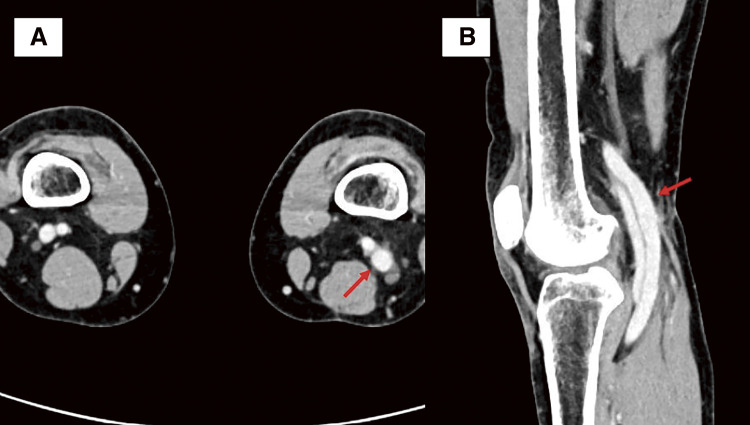
Fig. 3 Postoperative contrast-enhanced CT. (**A** and **B**) CT reveals no thrombus, coarctation, or other abnormalities. CT: computed tomography

A 59-year-old woman presented to our emergency department with a sudden loss of consciousness and CA. She had no history of heart or lung disease or DVT. One day prior to presentation, she experienced leg discomfort while working. She suddenly lost consciousness the following morning, and an ambulance was called. An agonal gasp was observed upon the arrival of the paramedics. Her blood pressure was unmeasurable, and her pulse was impalpable. Electrocardiographic waveforms revealed pulseless electrical activity. CA was diagnosed, and cardiopulmonary resuscitation (CPR) was initiated. Three minutes later, a return of spontaneous circulation was observed. The time from the family’s call for first aid to the arrival of the emergency medical services (EMS) team was 7 minutes, and the time from the arrival of the EMS team to CPR was 4 minutes. Resuscitation was successful before ambulance transport. On arrival at the hospital, the patient regained consciousness. An arterial blood gas showed the following: pH, 7.365; PaO_2_, 185 mmHg; PaCO_2_, 38.6 mmHg; HCO_3_, 21.5 mmol/L; ctHb, 12.9 g/dL; BE, –3.0 mmol/L; AG, 8.8 mmol/L; K+, 3.6 mmol/L; Lac, 3.3 mmol/L, and peripheral oxygen saturation (SpO_2_), 99% (on reservoir mask at 10 L per minute). Cardiac ultrasonography revealed right ventricular overload. Electrocardiography showed no acute ischemic changes and myocardial enzyme levels were normal. In addition, the patient’s serum D-dimer was significantly elevated (9000 μg/L). Based on these findings, we suspected a strong association between PE and CA. Since the patient’s general condition was stable, contrast-enhanced computed tomography (CT) was performed, revealing PE (**Fig. 1A**) and a left PVA (**Figs. 1B** and **1C**); therefore, the cause of PE was considered to be DVT originating from the PVA. The PE was a submassive lesion; the right side was occluded from the superior trunk pulmonary artery bifurcation, and the left side was occluded from the lingular artery bifurcation. In this successfully resuscitated case of CA, anticoagulation was administered, and thrombolysis was not performed because the patient’s hemodynamics were stable, and there was no evidence of right heart dysfunction. Anticoagulation treatment with a bolus dose of 5000 U of heparin was initiated, and heparin infusion was continuously administered. Furthermore, CT showed that the saccular PVA had a maximum diameter of 32 mm and a length of 30 mm. Because the risk of PE recurrence was high, surgery was deemed necessary. An evaluation of operative tolerance was conducted, and surgery was performed 16 days after diagnosis. Surgery was performed with the patient in the prone position, and the PVA was exposed using an L-incision on the posterior aspect of the popliteal fossa. A normal popliteal vein was identified proximally and distally, and traced back to the aneurysmal segment. A sharp demarcation was observed between the normal and abnormal portions of the vein. No intramural thrombosis was observed. Tangential aneurysmectomy and lateral venorrhaphy were performed (**Figs. 2A** and **2B**). The postoperative course was uneventful. After surgery, anticoagulation was re-started. Edoxaban 30 mg was administered and was continued until six months postoperatively. In addition, CT showed resolution of the pulmonary embolism six months postoperatively. One year later, no abnormal findings, such as thrombi or coarctation, were observed (**Figs. 3A** and **3B**).

## Discussion

Although dilatation of the termination of an incompetent short saphenous vein is occasionally seen, aneurysmal dilatation of the popliteal vein is rare.^3)^ The causal relationship is unclear but states that abnormal dilatation of deep veins is less frequent than varicosities of superficial veins. The frequency of PVAs has been estimated to be 0.2%–0.28% among patients with lower extremity venous disease, according to Zhao et al. in their report conducted at the Third Affiliated Hospital of Soochow University between March 2011 and August 2016.^4)^ In addition, PVA causing CA is rare. We searched PubMed for similar articles using “popliteal venous aneurysm” and “cardiac arrest” as search terms. However, only four references were extracted, and even among these, a few of them reported a similar clinical course, hence making the phenomenon rare. The pathogenesis of a PVA remains unknown. Trauma, inflammation, congenital weakness, and localized degenerative changes have been suggested as possible causes. A combination of congenital and acquired mechanisms is likely involved, in that PVAs may arise in congenitally predisposed patients as a consequence of mechanical or rheologic factors, or both.^5)^ However, in this case, not only were there no significant findings in the venous wall specimen submitted to pathology, but there were also no significant findings on CT that could suggest an acquired factor. Therefore, the pathogenesis of this case is also unknown. According to a review, 24%–51% of patients with a PVA present with PE.^2)^ In contrast, rupture is a rare complication of PVA.^6)^ A PVA does not present with specific signs or symptoms. Only 20% of the reported cases had a palpable mass in the popliteal fossa, making PVAs difficult to diagnose in asymptomatic patients.^7)^ In fulminant PE, up to 90% of CA occurs within 1–2 hours after the onset of symptoms.^1)^ Therefore, the presence of a PVA-causing PE is a life-threatening condition. Generally, surgical repair is indicated in all symptomatic patients, given the potential for serious thromboembolic complications. However, the management of asymptomatic PVAs remains controversial. Gabrielli et al. stated that preoperative contrast-enhanced CT is mandatory for patients in whom surgical repair of a PVA is considered in order to investigate the deep venous system and to define the venous anatomy.^6)^ In this case, PVA was incidentally diagnosed by contrast-enhanced CT and treated immediately, before PE recurred. Therefore, we recommend extending the contrast-enhanced CT to below the knees to investigate the cause of CA. An ultrasound is also useful to evaluate the popliteal vein, but it was not performed in this case. In our opinion, a contrast-enhanced CT reaching below the knee is important in determining the cause of CA to search for the presence of a PVA.

A previous report suggested that the diameter of a fusiform PVA is twice that of a normal popliteal vein, and one that is three times greater is critical.^2)^ Anticoagulation alone is not sufficient in patients with a PVA associated with thromboembolism such as PE. Patients who develop PE are at high risk of recurrent thromboembolism (80%) with anticoagulation alone and thus require surgical treatment of the PVA.^2)^ Various operative procedures have been used for venous reconstruction, and the incidence of complications varies greatly according to the operative method. Several surgical techniques are available for the treatment of PVAs, and aneurysm resection with preservation of venous continuity is recommended. The most common procedure is tangential aneurysmectomy, which is performed in 62%–78% of cases.^2^^,^^7)^ In particular, tangential aneurysmectomy with lateral venorrhaphy is the preferred technique because of its lower complication rate compared to that of other procedures. In contrast, resection of aneurysms with end-to-end anastomosis is not considered effective because it carries a high risk of early thrombosis.^8)^ In the present case, the PVA was dilated to three times the diameter of a normal vein and caused sudden CA; hence, tangential aneurysmectomy and lateral venorrhaphy were performed. When the PVA lumen was observed, it was possible to visually distinguish between the aneurysmal and nonaneurysmal venous walls. Therefore, for saccular aneurysms, the key is to make an incision on the side of the aneurysm while confirming its boundary. If there is little healthy vein wall remaining after the abnormal vein wall has been removed, another healthy vein would need to be transplanted to secure the lumen of the vein. If there is concern about stenosis, reconstruction using a small saphenous vein (SSV) patch should be considered. Therefore, exposing the SSV using the same incision is crucial. The angioplasty was performed using running sutures. A considerable resection of the dilated segment was performed while suturing the normal venous wall. Thus, to reduce the risk of postoperative stenosis or aneurysm, it is critical not to trim everything at once. In previous reports, postoperative anticoagulation management with warfarin was administered for 3–6 months.^4)^ In this case, there were no late complications, such as thromboembolism, recurrence of the venous aneurysm, or stenosis, six months after completion of postoperative anticoagulation therapy, but the short follow-up period and the unknown pathogenesis of PVA suggest that continued follow-up is important.

## Conclusion

A PVA is a rare pathological condition with potentially severe consequences. Therefore, although a PVA rarely causes CA, a thorough anatomical evaluation of the entire body should be performed when CA occurs. CT is crucial because it provides essential information, as in the present case. We performed surgical treatment of a PVA, which was discovered after further evaluation of sudden CA caused by PE. Regular follow-up is mandatory, and attention should be paid to late complications.

## Acknowledgments

We would like to thank Editage (www.editage.jp) for English language editing.

## Patient Consent Statement

Written informed consent was obtained from the patient to publish this case report.

## Disclosure Statement

The authors have no conflicts of interest to disclose.

## Author Contributions

Study conception: DS

Data collection: DS

Analysis: DS

Investigation: DS

Manuscript preparation: DS

Critical review and revision: all authors

Final approval of the article: all authors

Accountability for all aspects of the work: all authors.

## Abbreviations and Acronyms

PE: pulmonary embolism
CA: cardiac arrest
DVT: deep venous thrombosis
PVA: popliteal venous aneurysm
CPR: cardiopulmonary resuscitation
CT: computed tomography
SSV: small saphenous vein
